# Controlled outcome of Hirschsprung’s disease beyond adolescence: a single center experience

**DOI:** 10.1007/s00383-018-4391-5

**Published:** 2018-11-20

**Authors:** Elisabet Gustafson, Therese Larsson, Johan Danielson

**Affiliations:** 1 Institution of Women’s and Children’s Health, Uppsala University, Uppsala, Sweden; 2 Department of Pediatric Surgery, Akademiska Sjukhuset, 75185 Uppsala, Sweden

**Keywords:** Hirschsprung’s disease, Aganglionosis, Long-term, Bowel function, Quality of life

## Abstract

**Purpose:**

The aim of this study was to assess the function and quality of life of Hirschsprung’s Disease (HD) beyond adolescence and relate it to matched controls.

**Methods:**

All 203 patients diagnosed with HD at our department from 1961 to 1995 were identified. 21 had died, 43 had unclear diagnosis and 16 could not be traced. The remaining 123 patients were sent bowel function and SF-36 quality of life questionnaires. 69 patients (mean age 37.8, range 22–58, 13 female) responded and were matched with 138 age and sex-matched controls.

**Results:**

*Function*: HD-patients had significantly higher number of bowel movements per week, higher incidence of soiling, urgency, permanent stomas, use of laxatives, enemas and loperamide. HD-patients also scored significantly lower in their satisfaction with their bowel function. There was, however, no significant difference in Miller Incontinence score.

*QOL*: HD-patients reported a significantly higher incidence of negative impact by their bowel function on daily life, social interaction and ability to go on vacation. There were no significant differences in SF-36-scores.

**Conclusions:**

Bowel function has a lifelong negative impact on the lives of patients with HD. This strongly indicates a need for structured follow-up beyond adolescence.

## Introduction

Hirschsprung's disease (HD) is a congenital defect affecting the development of the enteric nervous system. This developmental defect results in the absence of enteric ganglia in the distal gut. The extent of this aganglionosis varies but is most frequently seen up to the level of the sigmoid colon. In rare cases the aganglionosis can affect the whole gastrostintestinal tract. The condition affects approximately 1/4500–5500 live births and is approximately three times more common in males [1, 2]. Over the years, the original surgical approaches designed by Swenson, Duhamel and Soave have been modified and today many centers use laparoscopic or totally transanal pull-through-procedures [3–8]. However, the majority of the HD-patients that today have reached adulthood have been operated with the older techniques. The outcome of HD in adulthood has been reported to be impaired and include both fecal incontinence and obstructive symptoms and the current knowledge has recently been summarized in a review article [9]. How functional outcome in adulthood differs from age-matched controls has, however, been sparsely investigated with only three studies to date [10–12]. All three publications found that functional outcome was worse when compared to controls. Two of these studies [11, 12] also investigated how bowel function affects the patient’s quality of life (QOL) with the GIQLI-questionnaire. In the publication by Järvi et al. [11], it was found that HD patients had marginally lower GIQLI-scores when compared to controls, whereas Granström et al. [12] found that HD patients had significantly lower GIQLI-scores.

The primary aim of this study was to evaluate the adult functional as well as QOL outcome of the HD-patients operated at our institution and relate this to the outcome of age matched controls.

## Methods

### Patients and controls

The case records and operative registry at the Department of Pediatric Surgery, University hospital, Uppsala, Sweden, were reviewed for all patients diagnosed with HD from 1961 to 1993 and clinical data were extracted. A total of 203 patients that had been diagnosed with HD were found. The charts of these patients were scrutinized to ensure the diagnosis. In 43 patients, the diagnosis of HD was incorrect or the diagnosis had not been confirmed with histopathology, and these patients were excluded from the study. At the time of follow-up 21 patients with HD had died. Another 16 patients could not be traced leaving a possible study population of 123 patients. These patients were sent invitations to participate as well as questionnaires. If the patients did not respond they were sent two reminders with a 6-week interval. Sixty-nine patients (56 percent) responded and were included in the study.

When the patient-group had been identified age and sex matched controls were identified from a database utilized in a previous study on anorectal malformations (ARM) [13]. These controls had been randomly selected from the National Swedish Population Register and had not undergone any kind of anorectal surgery. From the database two controls could be assigned to each patient leaving a control group of 138 controls.

There were no significant differences in demographic data between the HD- and the control group (Table 1).

**Table 1:** Demographic data for patients and controls

	HD	Controls	*P* value
*N*	69	138	N/A
Women/men	13/56	26/112	N/A
Age at follow-up, years	37.8 (35.5, 22–58.5)	37.4 (36, 19.5–65)	0.6684
Height, m	1.78 (1.8, 1.45–1,96)	1.79 (1.82, 1.53–1,98)	0.7775
Weight, kg	83.5 (85, 53–129)	84.1 (82 47–125)	0.7569
Body mass index	26 (25.8, 16.9–52.3)	26 (25.6, 19.1–36.1)	0.7792

Data are presented as frequencies or mean (median, range) as appropriate

### Assessment of patients and controls

The HD-patients and controls were assessed with a validated bowel function questionnaire [14], the Swedish version of Short form of the health survey (SF)-36 [15] and a non-validated questionnaire with questions that were not covered in the other questionnaires.

The validated bowel function questionnaire [14] consists of 49 questions relating to fecal incontinence, constipation and general bowel function symptoms, allowing calculation of [14] Miller’s incontinence score. This score is based on the type and frequency of incontinence episodes: zero represents total continence and 18 represents total incontinence [16]. The questionnaire also gives information of type of incontinence (classified as soiling, urge, non-urge or combination incontinence), medication, anal sensibility, deferring time and whether the anal continence affects social function in different ways.

SF-36 is a general QoL instrument not specifically designed to evaluate patients with colorectal disease. The Swedish SF-36 form is validated for the Swedish population [15, 17, 18].

The specific, non-validated, questionnaire included five questions regarding satisfaction of bowel function, urinary problems as well as the effects of bowel function on sexual function. It also included two additional questions for males regarding problems with erectile function and ejaculation. These questions had answers on a four-graded scale (1 = None, 2 = Some, 3 = Quite a lot, 4 = Very much).

### Statistical methods

Values are presented as proportions, means, medians and range. Fisher’s two-tailed exact-test was used to compare proportions and the Mann Whitney U-test was used for comparisons between groups. A *P* value below 0.05 was considered statistically significant. Statistica 12 software (StatSoft) was used for statistical analyses.

### Ethical considerations

The study was approved by the Regional Ethical Review Board (Dnr 2007/066). All patients and controls provided written informed consent.

## Results

### Bowel function

The HD patients reported significantly more bowel movements per week and were also less satisfied with their bowel function. There was a statistically higher incidence of stomas, urgency, soiling as well as frequent use of constipating medicines, laxatives and enemas in the HD-group. There were, however, no statistical differences in Miller incontinence score, ability to feel when they had a bowel movement, ability to discriminate between feces and flatus or use of protection in underwear between the HD-group and the control-group (Table 2).

**Table 2:** Parameters reflecting bowel function

	HD (*N* = 69)	Controls (*N* = 138)	*P* value
Satisfaction with bowel function (score 1–4, 4 = very satisfied)	3.0 (3, 1–4)	3.3 (4, 1–4)	**0.0070**
Number of bowel movements per week*	12.2 (10, 2–49)	8.3 (7, 2–15)	**0.0015**
Stomas	6 (8.7%)	0 (0%)	**0.0116**
Miller incontinence score (mean, median, range)	1.3 (0, 0–15)	0.8 (0, 0–8)	0.7575
Use of protection in underwear*	1 daytime (1.6%) 1 nighttime (1.6%)	0 daytime (0%) 0 nighttime (0%)	0.3186
Ability to feel when they need to defecate*	1 no (1.6%) 62 yes (98.4%)	1 no (0.7%) 137 yes (99.3%)	0.5389
Ability to discriminate between feces and flatus*	6 no (9.5%) 57 yes (90.5%)	14 no (10.1%) 124 yes (89.9%)	1.0000
Urgency when needing to defecate*	20 (31.7%)	25 (18.1%)	**0.0103**
Soiling*	10 (15.9%)	3 (2.2%)	**0.0008**
Use of loperamide or similar on a regular basis	5 (7.2%)	2 (1.4%)	**0.0348**
Use of laxatives on a regular basis	9 (13.0%)	2 (1.4%)	**0.0007**
Use of enemas on a regular basis*	9 (14.3%)	1 (0.7%)	**0.0002**

Results for satisfaction with bowel function, number of bowel movements and Miller incontinence score are presented as mean (median, range). Other parameters are presented as frequencies and percentages (within brackets). In variables marked with *patients with stomas have been omitted. *P* value < 0.05 is marked with bold font

### Urinary function and impact of bowel function on sexuality

The HD-group reported a statistically higher incidence of problems with urinary voiding but there was no difference in problems with urinary leakage. There were no statistical differences in the bowel function’s impact on either interest for or being able to take pleasure in sexual activities. Male patients reported no significant differences regarding erectile problems but had significantly more problems with ejaculation (see Table 3 for details).

**Table 3:** Parameters reflecting urinary and sexual function

	HD	Controls	*P* value
Problems with urinary voiding	1.3 (1, 1–3)	1.16 (1, 1–3)	**0.0230**
Problems with urinary leakage	1.2 (1, 1–4)	1.2 (1, 1–3)	0.6241
Bowel function has a negative impact on interest of sex	1.2 (1, 1–4)	1.1 (1, 1–4)	0.4836
Bowel function limits taking pleasure in sexual activity	1.2 (1, 1–4)	1.1 (1, 1–4)	0.8471
Problems with erection (males only)	1.2 (1, 1–2)	1.1 (1, 1–2)	**0.0770**
Problems with ejaculation (males only)	1.85 (1, 1–4)	1 (1, 1–1)	**0.0001**

These questions had answers on a four-graded scale (1 = None, 2 = Some, 3 = Quite a lot, 4 = Very much). Figures are reported as mean (median and range). *P* value < 0.05 is marked with bold font

### General questions of the function and the affect bowel function has on life in general

**Fig. 1:**
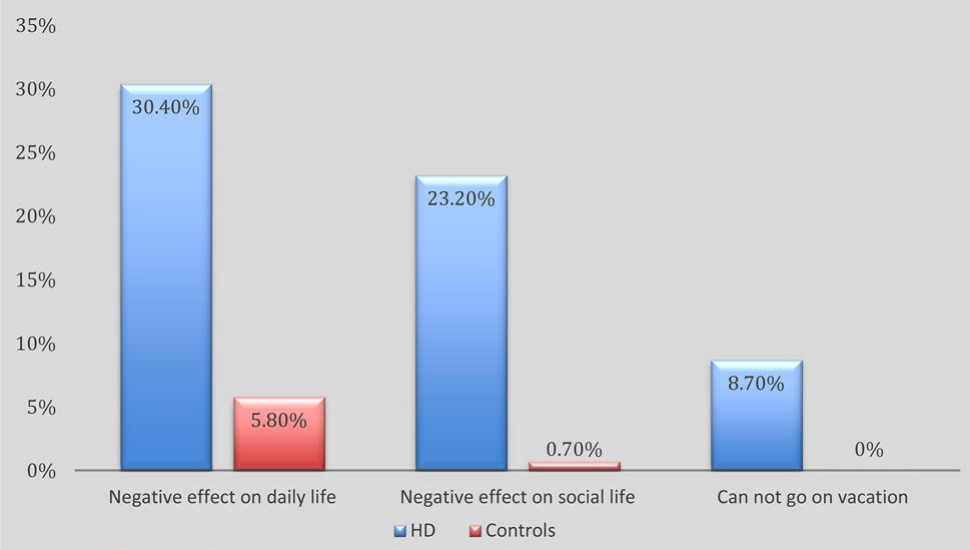
Graphical presentation of the reported incidence of how bowel function affects daily life, social life and ability to go on vacation. All categories had *P* < 0.05

Twenty-one HD patients stated that their bowel function had a negative impact on their daily lives. In comparison eight controls stated the same (*P* = 0.0000). Sixteen HD patients stated that their bowel function had a negative effect on their social life in comparison to only one control (*P* = 0.000). Six HD patients stated that they could not go on vacation due to their bowel function, whereas none of the controls stated the same (*P* = 0.0012). These figures are also presented as percentages in Fig. 1

### SF-36

**Fig. 2:**
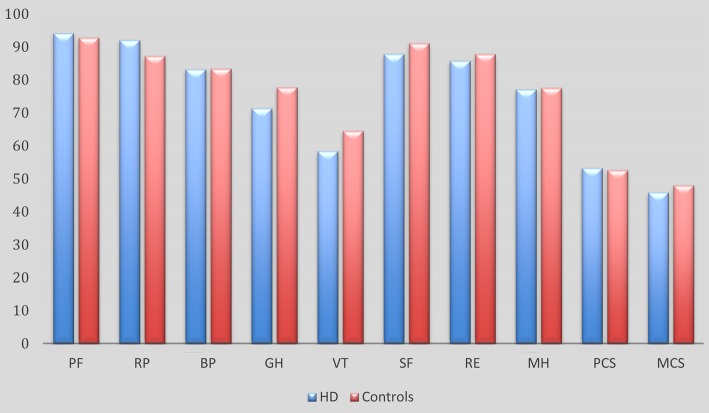
SF-36 results for HD-patients and their matched controls *PF* Physical Function, *RP* Role Physical, *BP* Bodily Pain, *GH* General Health, *VT* Vitality, *SF* Social Functioning, *RE* Role Emotional, *MH* Mental Health, *PCS* Physical Cluster Scale, *MCS* Mental Cluster Scale. No differences were significant

There were no statistically significant differences in any SF-36 parameters (Fig. 2).

### Drop out analysis

When comparing the included patients with the group that did not respond we found that the groups were similar but that the non-responders were somewhat, but not significantly, younger. The incidence of patients with Down’s syndrome was higher in the group of patients that did not respond. The difference was not significant.

## Discussion

The results from the present study show that adult patients operated for HD report significantly impaired bowel function. The incidence of symptoms consistent with both obstruction and fecal incontinence was higher among HD-patients compared to controls. This complies well with other recent studies of functional outcome of HD in adults compared to age- and sex-matched controls [10–12, 19]. These recent reports challenge the older view that HD has a favorable outcome in adulthood, exemplified by the Sherman report from 1989 [20].

Our outcome relating to QOL supports the findings of Granström et al. [12] that HD patients have impaired disease-specific, but normal generic, QOL when compared to age- and sex-matched controls. However, this was in contrast with the study of Neuvonen [19] where adult HD-patients scored lower on an emotional scale than the healthy controls.

We found that problems with bladder emptying and ejaculation were significantly more common among HD-patients than controls. Similar findings have been reported in adult patients by Ieri [21]. These findings might be attributed to the dissection close to the bladder neck and that it was standard at our institution to identify, dissect free and pull the spermatic duct to the side to avoid damage. This surgical maneuver may, in select cases, have had a potential to damage the spermatic duct.

The drop-out rate in this study was unfortunately quite high. However, the drop-out analysis implicated that our data are valid for the whole patient material.

Many pediatric surgeons have often taken to heart “the fact” that HD has a good long-term outcome, especially when compared to ARM. In a recently published study from our department we used the same questionnaires, as in this study, to evaluate adult patients with ARM [13]. In that study 34.5% of all ARM-patients reported that their bowel function had a negative effect on their daily life, 31% that it had a negative impact on their social life and that 7% could not go on vacation due to it. In comparison, 30.5% of the HD-patients in this study reported that their bowel function had a negative effect on their daily life, 23% that it had a negative effect on their social life and 8.5% could not go on vacation due to bowel-related problems. When looking at these figures it is clear that bowel function has a tremendous impact on the daily QOL of adult HD-patient. It may be time to reassess the view that adult HD-patients have an overall better outcome compared to ARM-patients. Järvi et al. [11] also showed that older age predicted a decline in bowel function. Moreover, constipation has also been observed to increase with rising age [22]. Taken together, these facts strongly implicate that there is a need for a structured follow-up of HD-patients into adolescence and beyond as suggested earlier in 2012 by Rintala and Pakarinen [22]. We believe that it is important that a dedicated team consisting of both pediatric surgeons and adult surgeons are involved in the follow-up of these patients. Such a setup will ensure transfer of knowledge over time and benefit both current and tomorrow’s patients.
